# Photocatalytic Phenylmethylamine Coupling Reaction of Organic–Inorganic Composites Based on Benzothiophene Polymers and TiO_2_

**DOI:** 10.3390/nano16060372

**Published:** 2026-03-19

**Authors:** Xin Li, Zhaozheng Yang, Lingyu Tai, Chengzhi Ma, Yuqing Hu, Jiawei Cai, Xin Shen, Pinghuai Liu, Lilin Tan, Yifan Chen

**Affiliations:** 1 Hainan Provincial Key Laboratory of Fine Chemicals, College of Chemical Engineering and Technology, Hainan University, Haikou 570228, China; 2 Laboratoire de Chimie Supramoléculaire, Institut de Science et d’Ingénierie Supramoléculaires (ISIS), Université de Strasbourg, 67000 Strasbourg, France; 3 Chemistry and Chemical Engineering Guangdong Laboratory, Shantou 515031, China

**Keywords:** polymer semiconductor, positive synergistic effect, organic–inorganic composites, photocatalytic coupling reaction, nitrogen-containing platform molecules

## Abstract

Benzothiophene polymers, as a class of novel organic semiconductor materials, exhibit significant potential in the field of photocatalysis due to their broad light-responsive range and tunable energy level structures. In this study, a benzothiophene-based polymer organic semiconductor (denoted as P42) was integrated with titanium dioxide (TiO_2_) via a simple sol–gel method, yielding an organic–inorganic hybrid material. This composite facilitates the modulation of energy level potentials and promotes the effective separation of photogenerated charges, thereby demonstrating remarkable synergistic catalytic performance in the photocatalytic oxidative coupling of benzylamines. By optimizing the ratio of organic to inorganic components and various photocatalytic reaction conditions, the hybrid material 1.7%P42-TiO_2_, containing 1.7 wt% of the dithiophene polymer without any metal cocatalysts, exhibited outstanding performance under an air atmosphere and visible light irradiation after 12 h. It achieved a yield of over 88.7% and a selectivity exceeding 89.8% in the synthesis of N-benzoylaniline, significantly surpassing the performance of pure TiO_2_ (52.9% yield, 54.9% selectivity) and P42 (54.4% yield, 54.9% selectivity). Structural and photophysical characterizations, including UV–Vis DRS, XRD, SEM, TEM, and EPR, reveal that the enhanced photocatalytic activity originates from broad visible-light absorption, improved charge separation, and well-matched energy levels. Mechanistic investigations suggest a synergistic pathway involving photoinduced hole oxidation and radical-mediated coupling. This work provides valuable insights and a reference for the solar-driven photocatalytic synthesis of nitrogen-containing platform molecules under mild conditions.

## 1. Introduction

Imines are critical synthetic intermediates for the synthesis of fine chemicals and pharmaceuticals [1,2]. The conventional preparation of imines through the condensation of amines and active carbonyl derivatives using Lewis acid/base catalysts not only generates serious chemical waste but also complicates post-processing, thereby resulting in environmental contamination [3,4]. Therefore, a critical solution lies in advancing green alternatives and transitioning to sustainable processes for complying with stringent environmental policies and minimizing ecological impact. In recent years, the photocatalytic oxidative coupling reaction of benzylamines, facilitated by advances in photocatalysis, has aroused significant interest [5,6]. The principal advantages of the photocatalytic benzylamine oxidation coupling reaction lie in its mild reaction conditions, environmental benignity, and high selectivity. This process utilizes light energy to drive the reaction under ambient temperature and pressure, employing air as a green oxidant instead of traditional toxic strong oxidizing agents, which effectively prevents pollution at the source. This method enables precise control over the oxidation process, facilitating the highly efficient and selective conversion of benzylamines into valuable imine chemicals. The materials employed in the photocatalytic oxidative coupling reaction of benzylamines primarily include inorganic semiconductors (e.g., TiO_2_) [7], polymer semiconductors (e.g., g-C_3_N_4_) [8], covalent organic frameworks (COFs) [9], metal–organic frameworks (MOFs) [10], polymer, as well as their various modified forms and heterojunction structures. Among them, organic polymer semiconductors have garnered considerable attention because their energy levels and bandgaps can be effectively adjusted through the introduction of functional groups, thereby enabling diverse photocatalytic reactions and significantly enhancing their activity [11]. Benzothiophene-based organic dyes or polymers have attracted significant interest owing to their excellent visible-light response capability and photochemical stability [12,13,14]; for example, a reported conjugated polymer incorporating benzothiophene-S, S-dioxide and two thiophene units. This polymer achieves a high HER activity of 20.31 mmol g^−1^·h^−1^ and a remarkable AQY of 7.04% at 500 nm [15]. Furthermore, Li et al. developed a cobalt-chelated copolymer photocatalyst (Co-PBDT-bpy) derived from benzodithiophene and pyridine. This composite exhibited a photocatalytic hydrogen evolution reaction (HER) rate of 140 μmol g^−1^·h^−1^ [16]. In our previous study, we constructed a hybrid organic–inorganic photocatalyst, denoted as P42-TiO_2_, comprising a benzothiophene-based polymer and titanium dioxide (TiO_2_) via the simple sol–gel method. This composite material exhibited a broad light-absorption range and well-matched redox potentials. Compared to the individual polymer and TiO_2_ components, the hybrid loaded with 1 wt% Pt nanoparticles demonstrated superior photocatalytic activity. The H_2_ generation rate for the composite exhibited a high activity of 745.0 μmol·g^−1^·h^−1^, which was a 38-fold enhancement to pure TiO_2_ material. Moreover, it is five times higher than that of a simple mechanical mixture of TiO_2_ doped with 1.7 wt% P42 (referred to as TiO_2_ + 1.7%P42), indicating the existence of a positively synergistic effect between the organic and inorganic semiconductors. These findings offer valuable insights for the rational design of photocatalytic materials [17]. Composited photocatalysis based on double or multiple functional materials has garnered considerable attention as an effective strategy for synthesizing imine-based fine chemicals utilizing benign oxidants such as oxygen or air [18,19]. The construction of organic–inorganic composite photocatalysts is particularly promising, as it enables the harnessing of complementary advantages from both components. The hybrids exhibit superior performance in visible-light utilization, tunable energy levels, and redox capabilities, thereby providing effective strategies to facilitate the separation and migration of charge carriers. A variety of synthesis methods are available for fabricating these composites, including deposition, coordination approaches, and sol–gel technique [20,21]. Among these, the sol–gel method is a simple and easy-to-operate process that can incorporate low-cost, environmentally benign titanium dioxide with high photocatalytic activity into organic polymer semiconductors, which could overcome the inherent limitations of organic polymer semiconductors, namely their hydrophobicity and poor dispersibility, requiring their photocatalytic reactions to be conducted in specific solvents and showing low reaction efficiency [22,23,24]. This integration results in the formation of uniform and stable organic–inorganic composite materials [25].

Here, building on our previous work, this study investigates the performance and mechanism of the photocatalytic benzylamine oxidative coupling reaction using an organic–inorganic composite , P42-TiO_2_, synthesized from a benz[1,2-b:4,5-b’]dithiophene polymer and titanium dioxide. The findings reveal that this photocatalytic system achieves notable performance under aerobic conditions without requiring metal cocatalysts. This research not only expands the application scope of polymer semiconductor materials in photocatalysis and provides a pathway for synthesizing nitrogen-containing organic molecules, but also establishes a theoretical foundation for understanding structure–activity relationships in such photocatalytic systems.

## 2. Materials and Methods

### 2.1. Materials

The chemical materials included benzo[1,2-b:4,5-b’]dithiophene polymer (P42), tetrabutyltitanate (Ti(OBu)_4_, 99%), acetic acid, tetrahydrofuran (THF), triethanolamine (TEOA, >99.0%), benzylamine, acetonitrile (99.9%), 1,4-benzoquinone, and silver nitrate (AgNO_3_), which were purchased from Allmers and Innochem Co., Ltd. (Beijing, China) and used without further purification.

### 2.2. Preparation of P42-TiO_2_ Composite Photocatalyst

The molecular structure of P42 and the preparation scheme for P42-TiO_2_ are presented in Figure S1. The synthesis procedure followed previously reported methods in the literature [17]. The composite was synthesized by combining 1.0 mL of tetrahydrofuran (THF), distilled water (0.106 mL), acetic acid (0.168 mL), and tetrabutyl titanate (1.0 mL) in a sample vial, which resulted in a transparent sol. Subsequently, the benzodithiophene-based polymer (P42) was dispersed in THF (1 mL) at different mass percentages (0, 0.43, 0.86, 1.7, and 2.5 wt%). Each dispersion was then introduced into the mixture. Upon addition, the mixture immediately transformed from a light-yellow transparent sol into a black gel. The product was aged overnight at 45 °C, yielding a black solid powder. The product was then purified by Soxhlet extraction for 48 h prior to being calcined at 200 °C. The calcination was performed with a temperature ramp rate of 2.25 °C/min. The resulting composites, prepared with different P42 loadings (0.43, 0.86, 1.7, and 2.5 wt% relative to TiO_2_ mass) , were designated as TiO_2_-200 and 0.43/0.86/1.7/2.5%P42-TiO_2_-200.

### 2.3. Characterization

UV–Vis spectra were recorded on a Lambda 750s spectrometer (PerkinElmer, Shelton, CT, USA). Using Cu-Kα_1_ radiation (λ = 1.54056 Å), PXRD patterns were recorded in Bragg–Brentano geometry using a Rigaku Smart Lab diffractometer (Tokyo, Japan). TEM, HRTEM, and EDX mapping were conducted on an FEI Talos F200X microscope (Hillsboro, OR, USA) functioning at 200 kV. SEM images were gained with a Hitachi SU8020 field-emission scanning electron microscope (Tokyo, Japan). The photocatalytic coupling reaction products and raw materials were quantified by gas chromatography (GC, FuLi GC9790Ⅱ, Zhejiang, China). XPS analysis was executed using a monochromated Al Kα source (Thermo Scientific Escalab 250Xi, Waltham, MA, USA) with a 650 μm spot size and a pass energy of 20 eV under ultrahigh vacuum.

### 2.4. Photocatalytic Studies

The reaction mixture containing benzylamine (BA) was placed in a 10 mL Schlenk tube and exposed to visible light under an air atmosphere to initiate the photocatalytic oxidative coupling. BA (0.2 mmol) and photocatalyst materials (5 mg) in acetonitrile (1.5 mL) were mixed and sonicated for 15 min to achieve homogeneity. The reaction system was then illuminated by a 300 W Xe lamp after passing through a 400 nm cut-off filter, providing an optical power density of approximately 1.1 W·cm^−2^. Several hours later, gas chromatography (GC) was used to analyze the supernatant following its filtration through a nylon syringe filter. The results are summarized in Table 1 and Table 2. BA conversion and the selectivity for N-benzylidenebenzylamine (N-BBA) were calculated using the equations below:

Conversion (%) = [(C_0_ − C_1_)/C_0_] × 100%

Selectivity (%) = [C_2_ × 2/(C_0_ − C_1_)] × 100%

where C_0,_ C_1_ and C_2_ represent the initial BA concentration and the concentrations of BA and N-BBA after the reaction, respectively, all quantifications were done with GC.

## 3. Results

**Figure 1:**
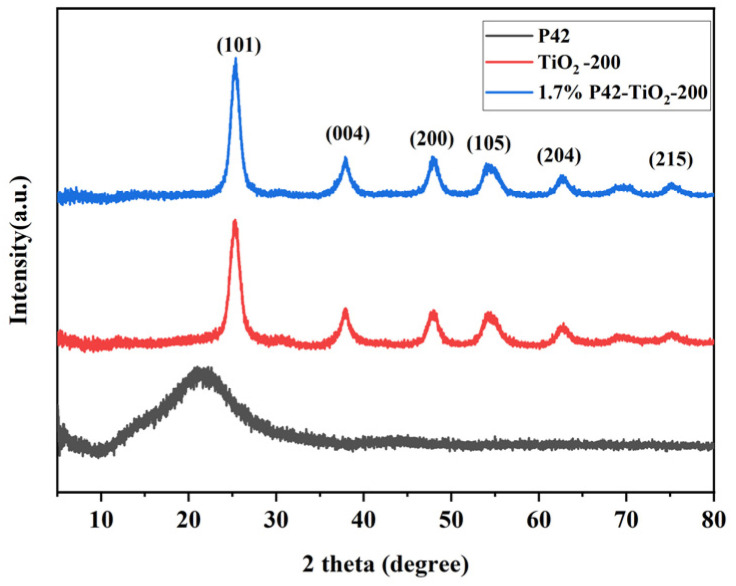
XRD patterns of 1.7%P42-TiO_2_-200 with different P42 content were recorded.

The synthesized materials were systematically characterized. Their crystal structure and components were subjected to powder X-ray diffraction (PXRD) and Fourier transform infrared (FTIR) spectroscopy. Their morphology was characterized by scanning and transmission electron microscopy (SEM and TEM). Their optical properties were analyzed using ultraviolet–visible diffuse reflectance spectroscopy (UV–Vis DRS). X-ray photoelectron spectroscopy (XPS) and the electrochemical workstation were employed to analyze photoelectrochemical properties. The XRD patterns of TiO_2_-200, pristine P42 and 1.7%P42-TiO_2_-200 are presented in Figure 1. Among them, TiO_2_-200 exhibits a pure anatase phase, as evidenced by its characteristic diffraction peaks at 2θ = 25.2°, 37.9°, 47.8°, 54.3°, and 62.5°. The pattern of the 1.7% P42-TiO_2_-200 composite is identical to that of the TiO_2_-200 precursor, indicating that the incorporation of P42 did not alter the crystal structure of TiO_2_. For the XRD pattern of composite, no distinct P42 diffraction signals were observed. This can be attributed to the low doping mass and high dispersion in the composite of P42. Furthermore, Fourier transform infrared (FTIR) spectroscopy of the 1.7% P42-TiO_2_-200 composite confirms the presence of P42 and TiO_2_ in the hybrid material. Figure S2 reveals the characteristic stretching vibrations associated with the aromatic C=C, thiophene C=C, C=S, and C=H groups in P42 are observed at 1500–1600 cm^−1^, 1000–1100 cm^−1^, 600–700 cm^−1^, and 2800–3000 cm^−1^, respectively. Meanwhile, the vibrational signatures corresponding to hydroxyl groups present on TiO_2_ were observed at approximately 3411 cm^−1^ and 1623 cm^−1^. Additionally, the Ti-O stretching vibration band of TiO_2_ was detected in the range of 446–798 cm^−1^. These results suggest that TiO_2_ interacts with P42 through a condensation reaction between hydroxyl and carbonyl groups. This confirms that the sol–gel method successfully integrated P42 with TiO_2_, forming a stable hybrid material.

**Figure 2:**
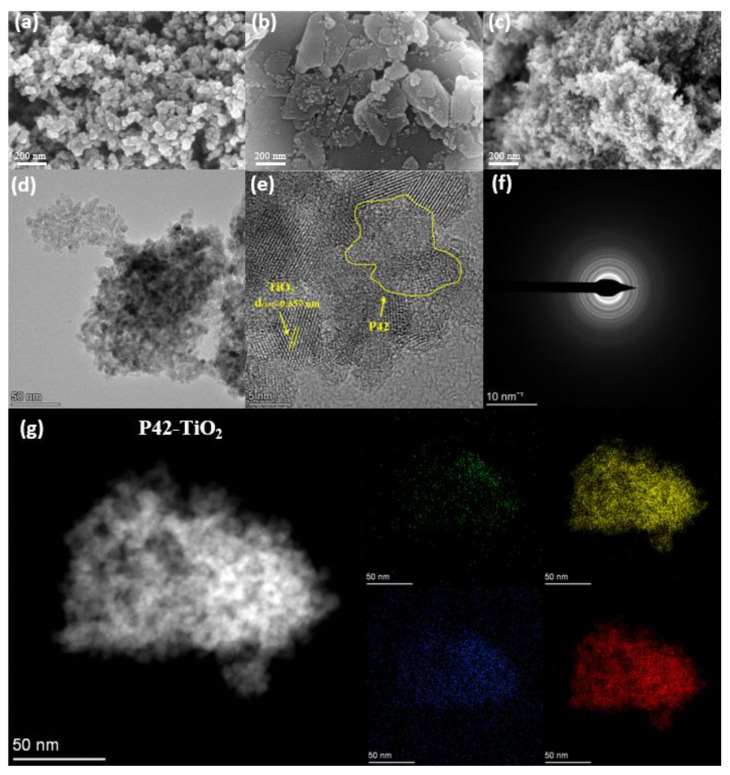
(**a**) HRSEM of TiO_2_, (**b**) HRSEM of P42, (**c**) HRSEM, (**d**) TEM, (**e**) HRTEM, (**f**) SAED and (**g**) the elemental mappings image of 1.7%P42-TiO_2_-200.

In Figure 2, SEM images show distinct morphological differences. In particular, the sample (Figure 2a) is composed of agglomerated spherical nanoparticles with an average diameter of about 30 nm of pure TiO_2_. P42 exhibits a predominantly amorphous planar microstructure, with sizes ranging from 50 to 300 nm (Figure 2b). In contrast, the morphology of the 1.7% P42-TiO_2_ composite (Figure 2c) is markedly different from that of pristine TiO_2_ and P42. It comprises elliptical nanospheres (∼10 nm in diameter) and small irregular nanoparticles. This observation indicates that the introduction of P42 alters the shape, size, and pore morphology of the original TiO_2_. Furthermore, HRTEM images and elemental mapping (Figure 2d) confirm this conclusion. And the distance of 0.357 nm observed between adjacent lattice stripes corresponds to the characteristic (101) reflection of anatase TiO_2_ (Figure 2e). The SAED results of the 1.7% P42-TiO_2_ composite nanomaterial confirm the anatase phase of TiO_2_. These findings are in agreement with the SAED data presented in Figure 2f. Furthermore, the corresponding elemental mapping (Figure 2g) and energy-dispersive X-ray (EDX) analysis (Figure S3) confirm the elements C, O, S, and Ti are distributed uniformly across the entire hybrid material.

**Figure 3:**
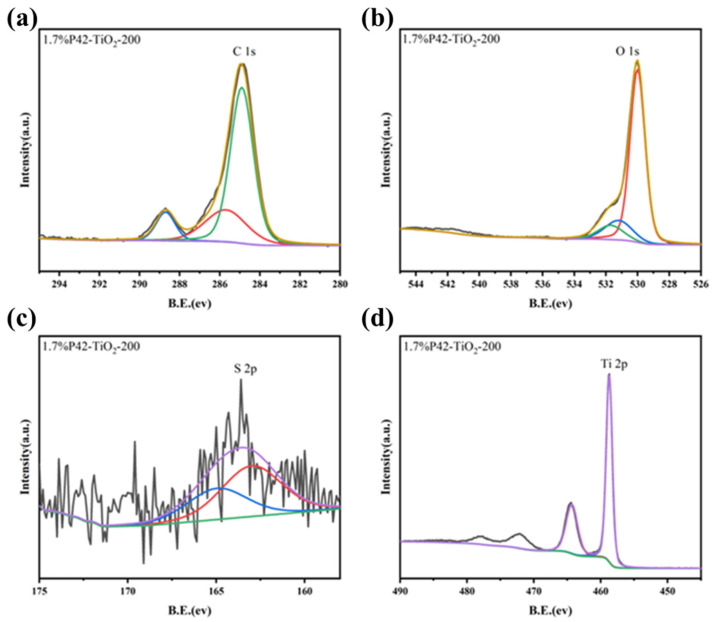
The XPS of the 1.7%P42-TiO_2_-200. (**a**)C 1s; (**b**) O 1s; (**c**)S 2p; (**d**) Ti 2p.

A comprehensive analysis of the XPS elemental fine-structure spectra indicates the formation of a distinct chemical bonding interface between P42 and TiO_2_. The characteristic Ti-O-C peak at 531.7 eV in the O 1s spectrum (Figure 3b), the positive shift (+0.2 eV) in the Ti 2p binding energy (Figure 3d), and the carboxyl carbon signal at 288.7 eV in the C 1s spectrum (Figure 3a) collectively suggest that P42 molecules coordinate with titanium atoms on the TiO_2_ surface via carboxyl (-COOH) and/or hydroxyl (-OH) functional groups, forming Ti-O-C covalent bonds. This chemical bonding not only ensures the stable immobilization of P42 on the TiO_2_ surface (no detachment after heat treatment at 200 °C), but also establishes an electron transport pathway: the positive shift in the Ti 2p binding energy indicates that P42 acts as an electron acceptor, facilitating the transfer of photogenerated electrons from the TiO_2_ conduction band to P42, thereby suppressing the recombination of photogenerated carriers. The attenuation of the sulfur signal at 163.0 eV in the S 2p spectrum (Figure 3c) indicates that the molecular structure of P42 remains intact during the formation of the composite material, as shown in Figure S4, which reveals a surface composition of 19.84% Ti, 59.25% O, 0.21% S, and 20.69% C. The thiophene/sulfide moieties may also contribute to the interfacial stability through π–π stacking and sulfur–metal interactions.

**Figure 4:**
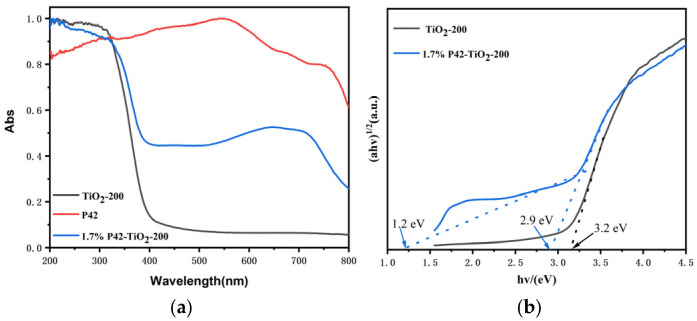
(**a**) The UV–Vis DRS of TiO_2_-200, 1.7%P42-TiO_2_-200 (**b**) The Tauc plot of TiO_2_-200 and 1.7%P42-TiO_2_-200.

The photophysical properties of P42, TiO_2_-200, and the 1.7% P42-TiO_2_-200 hybrid were investigated by solid-state UV–Vis spectroscopy (Figure 4a). TiO_2_-200 exhibits a characteristic absorption edge at 400 nm, corresponding to the bandgap of anatase TiO_2_ (3.2 eV) (Figure 4b). While the pristine P42 polymer displays broad absorption across 200–800 nm, consistent with its π-π* transitions and intramolecular charge-transfer character. Upon hybridization, the 1.7% P42-TiO_2_-200 composite exhibits a significantly broadened absorption profile from 200 to 800 nm. This profile comprises two distinct regions: an ultraviolet region (200–400 nm) originating from TiO_2_ and an enhanced visible-light absorption (400–800 nm) contributed by P42. This profile suggests strong electronic interaction between P42 and TiO_2_. The Tauc plot analysis (Figure 4b) shows that the hybrid material exhibits two distinct bandgaps (2.9 eV and 1.2 eV), which may be attributed to the role of P42 in modulating the bandgap of TiO_2_, as well as to the fact that P42 itself possesses a narrow bandgap. The Tauc for lot indicate (that 1.7% P42–TiO₂-200 possesses dual bandgaps, calculated to be 1.2 eV and 2.9 eV, corresponding to the P42 polymer and TiO_2_-200, respectively. The slight narrowing of the polymer’s bandgap from 1.3 eV (pristine P42, Figure S5) to 1.2 eV (in the hybrid) further indicates interfacial interactions between the components. Furthermore, the bandgap of TiO_2_-200 is 2.9 eV, representing a reduction compared to that of pristine anatase TiO_2_ (3.2 eV, Figure 4b). This redshift suggests interaction, such as interfacial coupling, between P42 and TiO_2_ in the composite.

**Figure 5:**
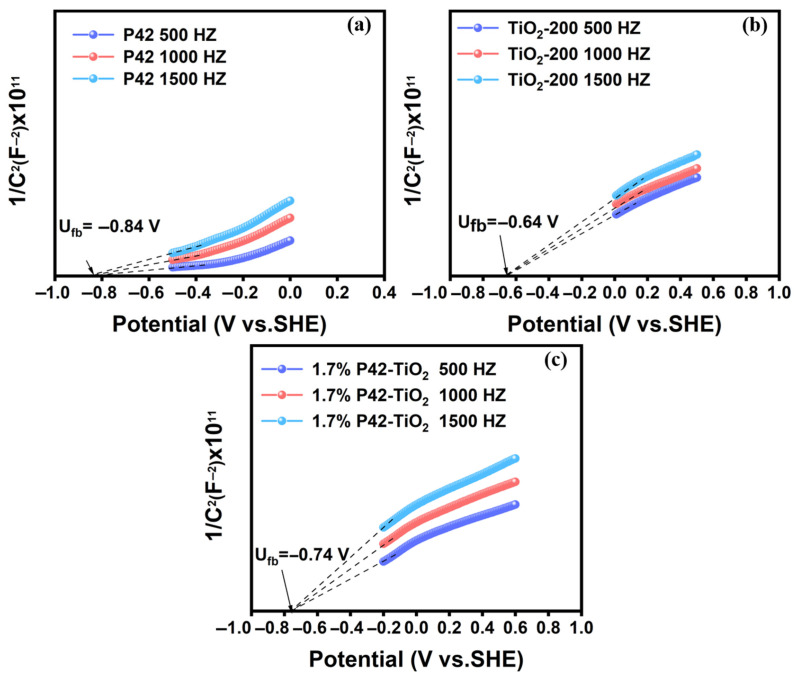
Mott–Schottky plot of (**a**) P42, (**b**) TiO_2_-200 and (**c**) 1.7%P42-TiO_2_-200.

It is well known that surface defects can strongly influence the properties of optical semiconductors. Through this influence, after photoexcitation, the hybrid material samples generate radicals via a specific mechanism, which may further alter their photocatalytic activity through a pronounced impact on their band structure. Hence, a series of photophysical and photoelectrochemical measurements were conducted to explore the origin of the enhanced photocatalytic activity. Mott–Schottky tests were performed at frequencies of 500, 1000, and 1500 Hz. As shown in Figure 5, the positive slopes of the obtained potential plots are consistent with typical n-type semiconductors. The flat-band potentials of TiO_2_-200, P42, and 1.7%P42-TiO_2_-200 versus the Ag/AgCl electrode were determined to be −0.64 eV, −0.84 eV, and −0.74 eV, respectively.It is widely accepted that the flat-band potential is 0.1–0.3 eV more positive than the conduction band potential in n-type semiconductors (0.2 eV adopted in this work). Therefore, the conduction band positions of TiO₂-200, P42, and 1.7% P42–TiO₂-200 were calculated to be −0.44 eV, −0.64 eV, and −0.54 eV (versus Ag/AgCl), all of which are more negative than the redox potential of O₂/·O₂⁻, rendering oxygen reduction thermodynamically feasible. Based on the bandgap diagram in Figure 4 and Figure S5, the valence band positions of TiO₂-200, P42, and 1.7% P42–TiO₂-200 were calculated as 2.76 eV, 0.66 eV, and 2.66 eV (versus Ag/AgCl), respectively, confirming the thermodynamic feasibility for benzylamine oxidation.

**Figure 6:**
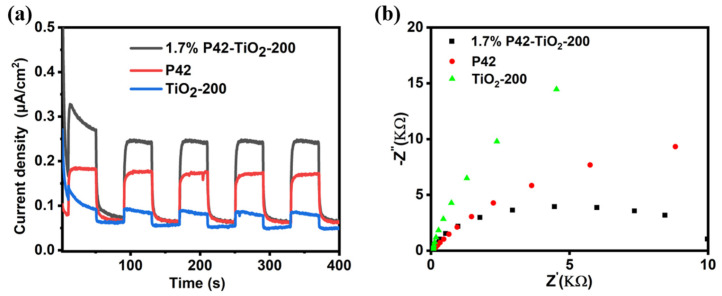
Photocurrent intensity (**a**) and EIS spectra (**b**) comparison of TiO_2_-200, 1.7%P42-TiO_2_-200 and P42.

As shown in Figure 6a, the photocurrent of 1.7% P42-TiO_2_-200 under visible light irradiation substantially exceeds that of P42 and TiO_2_, indicating markedly enhanced photogenerated charge separation efficiency when the catalyst is excited by visible light. These results reveal that the heterojunction formed between P42 and TiO_2_ accelerates the separation of e^−^/h^+^ pairs, thereby enhancing photocatalytic activity. Furthermore, as illustrated in Figure 6b, the 1.7% P42-TiO_2_-200 heterojunction exhibits a smaller arc radius in the electrochemical impedance spectrum compared to P42 and TiO_2_, indicating more efficient photogenerated charge separation.

**Figure 7:**
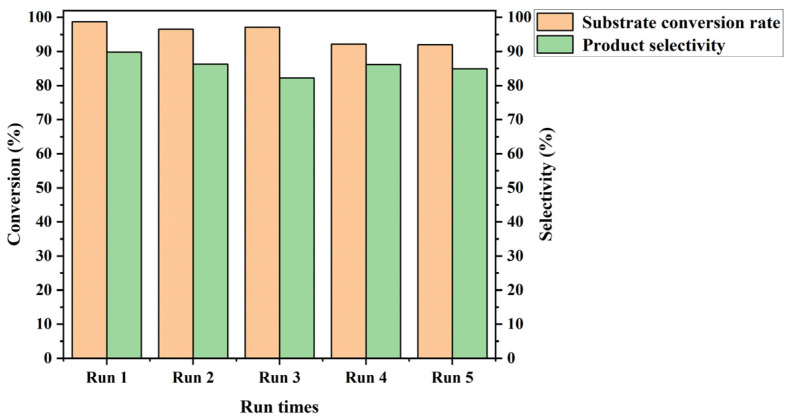
Conversion and selectivity of benzamine oxidation over the 1.7% P42-TiO_2_-200 photocatalyst.

Given its excellent light-harvesting ability and appropriate bandgap, the photocatalytic oxidative coupling reaction of BA in the P42-TiO_2_ composite was further evaluated under visible light and an air atmosphere. As presented in Table 1, all P42-TiO_2_ composites, regardless of their ratio, demonstrated highly efficient photocatalytic performance under identical conditions. A pronounced enhancement in N-BBA selectivity was observed as the P42 doping level was systematically adjusted from 0.43 wt% to 1.7 wt%. Among them, the 1.7% P42-TiO_2_-200 sample achieved the highest N-BBA conversion of 88.6%, with a BA conversion of 98.7%. (Table 1, entry 4). However, raising the P42 content further to 2.5 wt% led to a gradual drop in photocatalytic activity. (Table 1, entry 9). Whereas the pristine P42 and TiO_2_ components exhibited significantly lower performance (P42: 99.2% BA conversion, 54.9% N-BBA selectivity; TiO_2_: 91.4% BA conversion, 57.9% N-BBA selectivity; Table 1, entries 1–2), the superior activity of the hybrid material suggests that its specific electronic structure is likely a key contributing factor. The large planar conjugated system of the benzothiophene polymer, with its pronounced electron-donating capability, promotes the separation of photogenerated charges, thereby enhancing the catalytic conversion of BA. The photocatalytic performance of the 1.7% P42-TiO_2_ composite was further investigated as a function of calcination temperature. As presented in Table 1, the composites calcined at 100 °C and 200 °C exhibited high activity, with conversion/selectivity values of 99.0%/87.2% and 98.1%/99.9%, respectively (entries 3 and 4). However, at 300 °C, a significant decline in selectivity to 54.0% was observed despite the conversion remaining high at 98.9% (entry 5). A more pronounced deterioration occurred at 400 °C, where both conversion and selectivity dropped markedly to 71.0% and 51.3%, respectively (entry 6). These results demonstrate that high calcination temperatures adversely affect catalytic performance. This deleterious effect is likely attributable to the degradation of the conjugated structure of the benzothiophene polymer, which can lead to diminished light absorption, carbonization, and the loss of electron-donating capacity. Thus, P42 serves as a pivotal functional regulator in the hybrid material, not only broadening the spectral response as a photosensitizer but also promoting charge separation as an organic semiconductor/electron accumulator. The practical relevance of this stable architecture was demonstrated via cyclic testing, wherein the 1.7% P42-TiO_2_ composite maintained high conversion efficiency and selectivity toward N-BBA over five consecutive runs (Figure 7), thereby confirming its excellent reusability.

**Figure 8:**
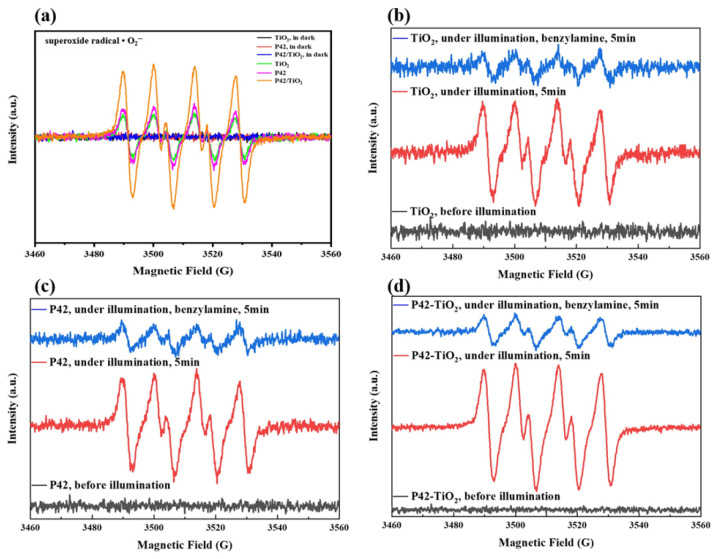
Electron paramagnetic resonance images of superoxide radical capture in P42, TiO_2_ and P42-TiO_2_ catalytic systems in air atmosphere were obtained, (**a**) superoxide radical intensity signals produced by P42, TiO_2_ and P42-TiO_2_ catalytic systems after 5 min each of dark and light; (**b**) the intensity signal of superoxide radicals produced by TiO_2_ catalytic system in the dark after 5 min of illumination and after 5 min of illumination with benzylamine; (**c**) the intensity signal of superoxide radicals produced by P42 catalytic system in the dark and light for 5 min, and in the light with benzylamine for 5 min; (**d**): the intensity signal of superoxide radicals produced by P42-TiO_2_ catalytic system in the dark and light for 5 min and in the light with benzylamine for 5 min.

In order to identify the key reactive intermediates, quenching experiments were conducted, and the detailed outcomes are presented in Table 2. The severe inhibition of imine formation in the presence of AgNO_3_ (an electron scavenger, yielding 10.6%; Entry 2) confirms photogenerated electrons as crucial. The similarly suppressed yields with scavengers for ·O_2_^−^ (benzoquinone, BQ, 16.9%; Entry 3) and ^1^O_2_ (α-terpineol, 25.7%; Entry 4) further suggest the involvement of these reactive oxygen species. The partial inhibition observed with the hole scavenger KI (16.9%; Entry 5) the participation of holes (h^+^), which are likely involved in the substrate oxidation step. The minimal inhibition observed with a ·OH scavenger (79.7% yield; Entry 6) rules out a significant role for hydroxyl radicals, which is consistent with energy level considerations. This result supports the proposal that superoxide (·O_2_^−^) and singlet oxygen (^1^O_2_) act in concerted yet distinct roles as the primary reactive oxygen species (ROS), indicating a more complex mechanistic network than a single-pathway reaction. Previous studies have established that molecular oxygen (O_2_) plays an essential role in the photo-oxidative coupling of benzylamine (BA) to N-benzylidenebenzylamine (N-BBA). To further elucidate the involvement of oxygen-derived intermediates, we performed electron paramagnetic resonance (EPR) studies . Negligible EPR signals were observed for P42, TiO_2_ and their composite under dark conditions (Figure 8a). Upon visible-light irradiation, however, the P42-TiO_2_ composite, using DMPO as a spin trap , exhibited a distinct EPR signal characteristic of superoxide radical anions (•O_2_^−^). Under the same conditions, both P42 and TiO_2_ alone showed markedly weaker signal intensities, indicating a substantially higher yield of •O_2_^−^ generation in the P42-TiO_2_ hybrid material. Furthermore, in the individual TiO_2_ and P42 systems, superoxide radical signals were detected after 5 min of illumination but weakened upon the introduction of benzylamine (Figure 8b,c), suggesting that BA effectively scavenges •O_2_^−^. In the P42-TiO_2_ system, the strong •O_2_^−^ signals under illumination were similarly quenched by benzylamine (Figure 8d). This observation further confirms the superior •O_2_^−^ generation capability of the composite.

These findings collectively demonstrate that the P42-TiO_2_ composite exhibits a significantly enhanced capacity for superoxide radical generation under visible light. This enhancement is attributed to the synergistic interaction between P42 and TiO_2_. The benzothiophene-based polymer imparts efficient photoinduced charge separation and interfacial electron transfer, enabling the photoreduction of O_2_ to active •O_2_^−^ species. Thus, the synergistic mechanism in the P42-TiO_2_ composite paves the way for its application in efficient photocatalytic processes.

**Scheme 1:**
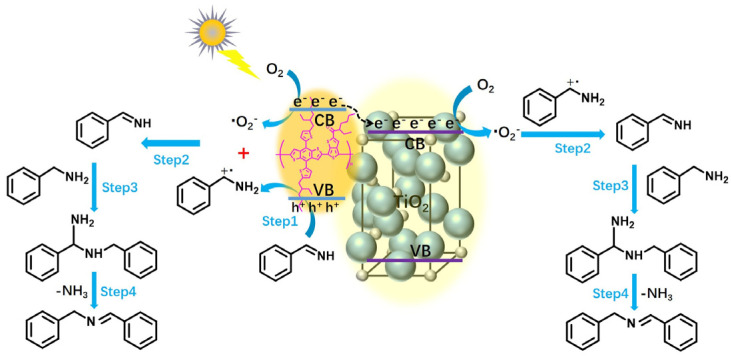
Proposed mechanism of photocatalytic coupling oxidation of P42-TiO_2_ over visible-light irradiation.

In light of these findings, Scheme 1 outlines a proposed photocatalytic mechanism for imine formation under aerobic conditions. Upon visible-light irradiation, the photosensitizer P42 is excited, thereby generating electron–hole pairs and migrating to the TiO_2_ conduction band, reducing adsorbed O_2_ to generate superoxide radicals (•O_2_^−^) and thereby achieving efficient charge separation. Concurrently, the holes in the P42 valence band oxidize benzylamine, forming benzylamine cation radicals (Step 1). The resulting cation radical subsequently reacts with the •O_2_^−^ species, forming the aldimine intermediate (Ph-CH=NH) in Step 2. Subsequently, this intermediate condenses with another benzylamine molecule to generate an N-benzyl-1-phenylmethane diamine intermediate (Step 3). Then the diamine intermediate undergoes oxidative dehydrogenation, catalyzed by a hole, to afford the target imine product, N-benzylidenebenzylamine (N-BBA) (Step 4).

## 4. Conclusions

In conclusion, a benzothiophene polymer-TiO_2_ organic–inorganic hybrid was synthesized via a facile sol–gel method. Comprehensive characterization confirmed the successful integration of the polymer within the composite. Among the tested variants, the P42-TiO_2_ composite demonstrated superior photocatalytic activity, attaining 98.7% conversion and 89.8% selectivity toward N-benzylidenebenzylamine (N-BBA), significantly outperforming pure TiO_2_ and P42 counterparts. This enhanced performance is attributed to improved visible-light absorption, efficient charge separation and transport, and a distinct synergistic effect between P42 and TiO_2_ in the hybrid. Despite limited exploration of polythiophene-based polymers in photocatalytic amine coupling reactions, this study establishes benzodithiophene-based polymers as sustainable, metal-free alternatives to conventional noble-metal catalysts. These materials open new avenues for the synthesis of nitrogen-containing fine chemicals and underscore the potential of carbon-based systems in addressing resource constraints in photocatalysis.

## Figures and Tables

**Table 1:** Photocatalytic oxidation of BA to N-BBA under various conditions.

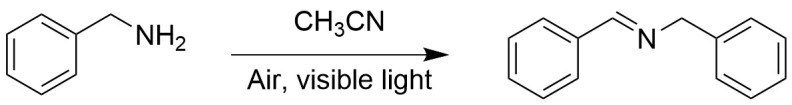			
Entry	Catalysis	Conv. (%)	Select. (%)
1	P42	99.2	54.9
2	TiO_2_	91.4	57.9
3	1.7% P42-TiO_2_–100 °C	99.0	87.2
4	1.7% P42-TiO_2_–200 °C	98.7	89.8
5	1.7% P42-TiO_2_–300 °C	98.9	54.0
6	1.7% P42-TiO_2_–400 °C	71.0	51.3
7	0.43% P42-TiO_2_–100 °C	99.3	34.7
8	0.86% P42-TiO_2_–100 °C	99.2	53.7
9	2.5% P42-TiO_2_–100 °C	99.3	69.9

Reaction conditions: BA (0.2 mmol), catalyst (5 mg), CH_3_CN (1.5 mL), Air (1 atm), λ > 400 nm, 12 h.

**Table 2:** Experimental results of 1.7% P42-TiO_2_-200 benzylamine oxidative coupling free radical quenching.

Entry	Catalysis	Atmosphere	Quenching Agent	Quenching Object	Yield. (%)
1	1.7% P42-TiO_2_–200 °C	Air	-	-	88.7
2	1.7% P42-TiO_2_–200 °C	Air	AgNO_3_	Electron(e^-^)	10.6
3	1.7% P42-TiO_2_–200 °C	Air	BQ	Superoxide radicl (**•**O_2_^−^)	16.9
4	1.7% P42-TiO_2_–200 °C	Air	α-Terpineol	Singlet oxygen (^1^O_2_)	25.7
5	1.7% P42-TiO_2_–200 °C	Air	KI	Hole	16.9
6	1.7% P42-TiO_2_–200 °C	Air	Tert-butylalcohol	Hydroxyl Radical (**•**OH-)	79.7

Reaction conditions: BA (0.2 mmol), catalyst (5 mg), Quenching agent (1 mM) CH_3_CN (1.5 mL), Air (1 atm), λ > 400 nm, 12 h.

## Data Availability

The original contributions presented in this study are included in the article/Supplementary Material. Further inquiries can be directed to the corresponding authors.

## Supplementary Materials

The following supporting information can be downloaded at: https://www.mdpi.com/article/10.3390/nano16060372/s1, Figure S1. The preparation process of the 1.7%P42-TiO_2_-200 material. Figure S2. FT-IR spectra of TiO_2_-200 and series of P42-TiO_2_-200 hybrid samples. Figure S3. The EDX of 1.7%P42-TiO_2_-200. Figure S4. Atmoic Fraction of 1.7% P42-TiO_2_-200 °C. Figure S5. The tauc plot of P42.
