# Supplementary material for: Photocatalytic Phenylmethylamine Coupling Reaction of Organic–Inorganic Composites Based on Benzothiophene Polymers and TiO2
Source: Nanomaterials (Basel). 2026 Mar 19;16(6):372. doi: 10.3390/nano16060372 (PMC13029632; doi:10.3390/nano16060372)
Supplement: Supplementary file 1 [file nanomaterials-16-00372-s001.zip › nanomaterials-4197918-supplementary.pdf]

Supporting Information

Photocatalytic byramine oxidation by organic-inorganic hybrid materials based on benzothiophene polymers and TiO_2

Xin Li [1], Chengzhi Ma[1], Yuqing Hu[1], Jiawei Cai[1], Xin Shen[1], Pinghuai Liu[1], Yi-Fan Chen [1,*] Li-Lin Tan[2,*]

1 Hainan Provincial Key Laboratory of Fine Chemicals, College of Chemical Engineering and Technology, Hainan University, Haikou 570228, China
2 Chemistry and Chemical Engineering Guangdong Laboratory, Shantou 515031, China
* Correspondence: chenyifan@hainanu.edu.cn (Y.C.); tanll@ccelab.com.cn (L.T.)

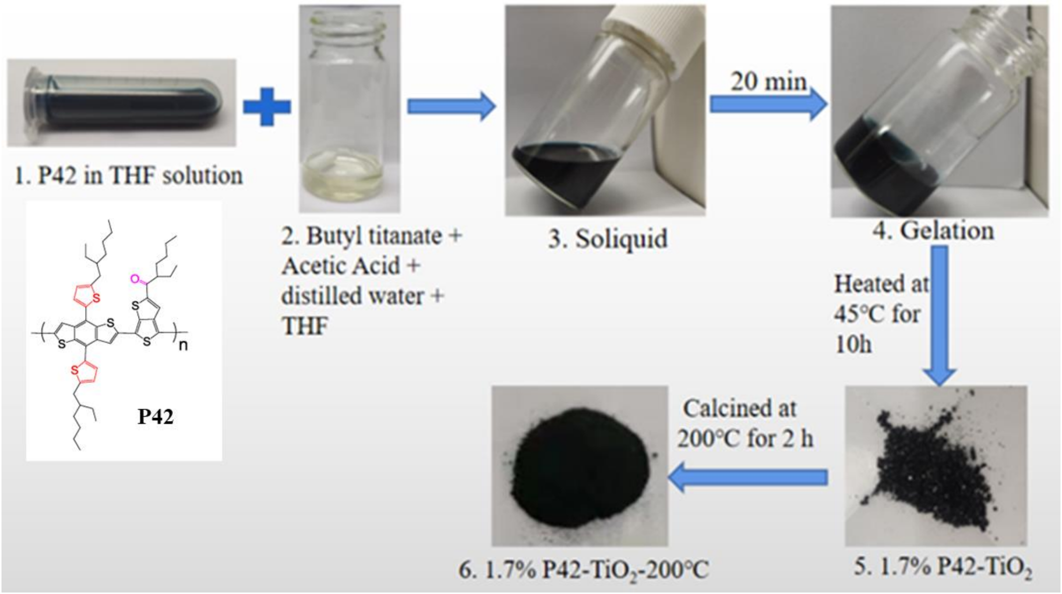

Figure S1. The preparation process of the 1.7%P42-TiO_2-200 material.

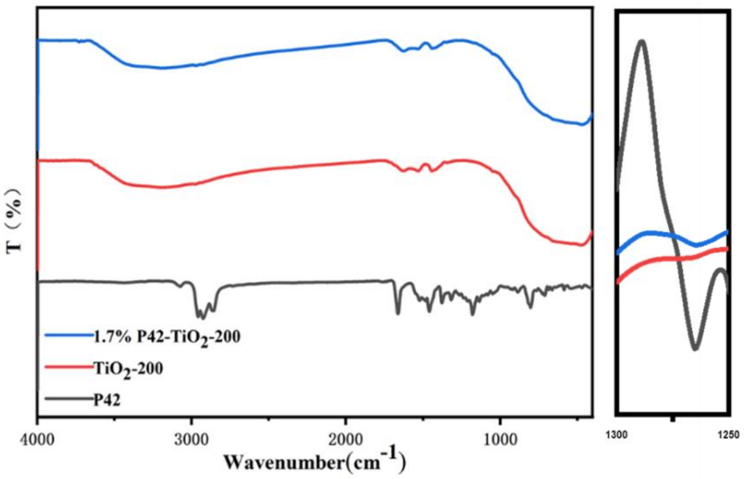

Figure S2. FT-IR spectra of TiO_2-200 and series of P42-TiO_2 -200 hybrid samples.

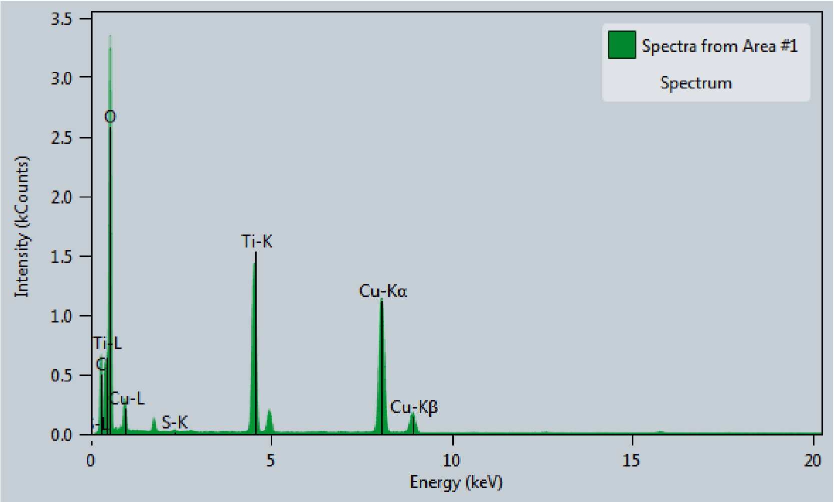

Figure S3. The EDX of 1.7%P42-TiO_2-200

Z	Element	Family	Atomic Fraction (%)	Atomic Error (%)	Mass Fraction (%)	Mass Error (%)	Fit error (%)
6	C	K	20.69	4.05	11.54	1.27	2.95
8	O	K	59.25	16.52	44.03	10.00	1.80
16	S	K	0.21	0.06	0.31	0.07	2.61
22	Ti	K	19.84	4.65	44.12	7.49	0.11

Figure S4. Atmoic Fraction of 1.7% P42-TiO_2 -200℃.

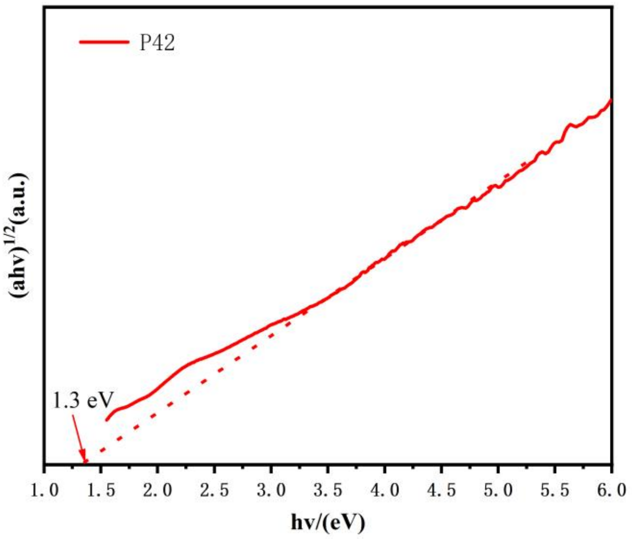

Figure S5. The tauc plot of P42.
